# Brief Report: Cystatin C Provides Substantially Higher Glomerular Filtration Rate Estimates Than Creatinine in a Subset of Black People With HIV on Current Antiretroviral Regimens

**DOI:** 10.1097/QAI.0000000000003555

**Published:** 2024-05-11

**Authors:** Lourdes Dominguez-Dominguez, Lisa Hamzah, Julie Fox, Royce P. Vincent, Frank A. Post

**Affiliations:** a King's College Hospital NHS Foundation Trust, London, United Kingdom;; b Berkshire Healthcare NHS Foundation Trust, Slough, United Kingdom;; c St George's Hospital NHS Foundation Trust, London, United Kingdom;; d Guy's and St Thomas' Hospital NHS Foundation Trust, London, United Kingdom;; e King's College London, London, United Kingdom; and; f Department of Clinical Biochemistry (Synnovis), King's College Hospital NHS Foundation Trust, London, United Kingdom.

**Keywords:** HIV, eGFR, kidney, CKD, creatinine, cystatin C

## Abstract

**Background:**

In African populations, estimated glomerular filtration rate by cystatin C (eGFRcys) is better aligned with gold-standard GFR measurements than eGFR by creatinine (eGFRcr). Moreover, eGFRcys is unaffected by the effects of antiretroviral therapy (ART) on tubular secretion and may thus provide better estimates of GFR in people with HIV on ART.

**Setting:**

Observational cohort study of people of African ancestry living with suppressed HIV RNA on ART in London, United Kingdom.

**Methods:**

Cross-sectional analysis of 360 paired serum creatinine and cystatin C measurements. Participants whose eGFRcys substantially (>10%) exceeded eGFRcr were identified, and factors associated with this outcome were identified in logistic regression analysis.

**Results:**

The median age of participants was 52 years, 56% were women, and 82% born in Africa or the Caribbean. The eGFRcys substantially exceeded eGFRcr in 42% of participants in the overall cohort, and in 68% of those with eGFRcr 45–75 mL/min/1.73 m^2^. In multivariable analysis, a higher eGFRcr was associated with lower odds (0.59 [0.50, 0.68] per 10 mL/min/1.73 m^2^ increase) of eGFRcys substantially exceeding eGFRcr; a higher BMI was also associated with this outcome, while ART regimens inhibiting tubular secretion of creatinine were not predictive. Of the 22 participants with eGFRcr 45–60 mL/min/1.73 m^2^, 16 (73%) had eGFRcys >60 mL/min/1.73 m^2^.

**Conclusions:**

We report substantially higher eGFRcys than eGFRcr in a subset of people of African ancestry with suppressed HIV, particularly among those with eGFRcr 45–75 mL/min/1.73 m^2^. In this population, eGFRcys provides clinically useful information irrespective of ART regimen.

## Abstract 2

Supplemental Digital Content is Available in the Text.

## INTRODUCTION

Chronic kidney disease (CKD) is a major complication of HIV infection and disproportionally affects people of African ancestry.^1^ The high prevalence of CKD in these populations may result from immunodeficiency, viral replication and immune activation, adverse effects of antiretroviral therapy (ART) on the kidney, comorbid conditions, and genetic predisposition, for example, variants of apolipoprotein L1 (APOL1) and sickle cell trait.^2^ In addition, several antiretrovirals including dolutegravir and bictegravir inhibit tubular secretion of creatinine, resulting in higher plasma concentrations and lower estimates of glomerular filtration rate (eGFR).^3,4^ Hence, monitoring of kidney function is an integral part of HIV care; although creatinine is routinely used for this purpose, interpretation of creatinine-based estimates of GFR is not entirely straightforward.

Cystatin C can also be used to provide estimates of kidney function. Cystatin C is not subject to tubular secretion and, therefore, a direct marker of GFR. Estimates of GFR calculated with cystatin C (eGFRcys) are best aligned with gold standard (iohexol) measurements of GFR in sub-Saharan African populations^5^ and less biased than creatinine-based eGFR estimates (eGFRcr) in people with HIV; they may also provide a better assessment of kidney function in those on antiretroviral therapy that affects tubular creatinine secretion.^6,7^ In the general population, approximately 30% have substantially higher eGFRcys values than eGFRcr, and Black ethnicity is a predictor of eGFRcys being greater than eGFRcr.^8^ Those with an eGFRcys greater than eGFRcr experience fewer serious adverse outcomes such as mortality, cardiovascular events, and end stage kidney disease.^9–12^

We sought to describe the proportion and clinical characteristics of individuals with HIV of African or Caribbean ancestry for whom eGFRcys was substantially greater (>10%) than eGFRcr, with a particular focus on those with eGFRcr 45–75 mL/min/1.73 m^2^ for whom a revised GFR estimate may exclude a CKD diagnosis.

## METHODS

We used paired serum creatinine and cystatin C, and urinary protein and albumin measurements from participants of the cardiovascular disease, kidney disease, and diabetes in people of African ancestry (CKD-AFRICA) study, a cross-sectional substudy of the Genetic Determinants of Kidney Disease in people of Africa Ancestry with HIV (GEN-AFRICA study; NCT05685810).^13^ The CKD-AFRICA study enrolled Black people with HIV aged 30–65 years at 3 sites in south London, United Kingdom, between September 2020 and January 2022.^14,15^ All participants provided written informed consent. For each participant, we calculated eGFRcys (CKD-EPI 2012) and eGFRcr (CKD-EPI 2021), and urine protein/creatinine and albumin/creatinine ratios (ACR); analyses were restricted to participants with plasma HIV RNA <200 copies/mL.^15^

We stratified participants into 3 groups: those for whom (1) eGFRcys was substantially (>10%) higher than eGFRcr, (2) eGFRcys was within 10% of the eGFRcr, and (3) eGFRcys was substantially (>10%) lower than the eGFRcr. We used logistic regression analysis to identify factors associated with eGFRcys >10% higher than eGFRcr, with the other 2 groups combined as referent; factors associated with this outcome in univariable analysis (*P* < 0.1) were included in the multivariable models.

## RESULTS

We included 360 participants with a median age of 52 years; 44% were men and 82% were born in sub-Saharan Africa or the Caribbean. Most had longstanding HIV and all were on ART, with a median CD4 cell count of 551 cells/mm^3^; 21% were on regimens containing efavirenz or nevirapine (which do not affect creatinine secretion), 21% on regimens containing bictegravir or dolutegravir (which have greatest effect on creatinine secretion), and 54% on other third agents with variable effect on creatinine secretion (Table 1). The median eGFRcr was 85 (interquartile range 70–100) mL/min/1.73 m^2^ and the median eGFRcys was 89 (75–104) mL/min/1.73 m^2^.

**TABLE 1.** Clinical Characteristics of the Study Participants

		Overall (N = 360)	eGFRcys C < eGFRcr (N = 68)	eGFRcys C = eGFRcr (N = 141)	eGFRcys C > eGFRcr (N = 151)	*P*
Demographic and HIV data						
Age	Median [IQR]	52.1 [45.2–57.4]	53.2 [45.4–58.9]	51.7 [45.2–56.8]	51.8 [45.0–56.9]	0.28
Gender (male)	N (%)	159 (44.2)	20 (29.4)	62 (44.0)	77 (51.0)	0.012
Born in Africa/Caribbean	N (%)	295 (81.9)	54 (79.4)	116 (82.3)	125 (82.8)	0.83
Time since HIV diagnosis (yr)	Median [IQR]	14 [9–18]	15 [10–19]	13 [10–18]	14 [9–19]	0.66
Time since starting ART (yr)	Median [IQR]	11 [7–15]	12 [7–17]	10 [7–15]	10 [7–14]	0.37
TDF-based ART regimen	N (%)	127 (35.3)	26 (38.2)	57 (40.4)	44 (29.1)	0.11
ART regimen by 3rd agent	N (%)					0.004
Efavirenz or nevirapine		75 (20.8)	9 (13.2)	31 (22.0)	35 (23.2)	
Rilpivirine		43 (11.9)	5 (7.4)	20 (14.2)	18 (11.9)	
Ritonavir-boosted PI		30 (8.3)	8 (11.8)	12 (8.5)	10 (6.6)	
Cobicistat-boosted PI		64 (17.8)	10 (14.7)	31 (22.0)	23 (15.2)	
Raltegravir		58 (16.1)	18 (26.5)	22 (15.6)	18 (11.9)	
Bictegravir or dolutegravir		75 (20.8)	11 (16.2)	21 (14.9)	43 (28.5)	
Other		15 (4.2)	7 (10.3)	4 (2.8)	4 (2.6)	
Nadir CD4 cell count	Median [IQR]	161 [71–278]	151 [61–260]	157 [70–268]	172 [74–292]	0.37
Current CD4 cell count	Median [IQR]	551 [376–751]	451 [296–680]	562 [395–756]	593 [405–771]	0.032
Risk factors and comorbidities						
Smoking status	N (%)					0.81
Nonsmoker		278 (80.6)	55 (85.9)	108 (78.8)	115 (79.9)	
Ex-smoker		34 (9.9)	4 (6.2)	15 (10.9)	15 (10.4)	
Current smoker		33 (9.6)	5 (7.8)	14 (10.2)	14 (9.7)	
CRP (mg/L)	Median [IQR]	2.0 [1.0–5.0]	4.0 [2.3–9.0]	2.0 [1.0–5.0]	2.0 [1.0–4.0]	<0.001
BMI (kg/m^2^)	Median [IQR]	30.2 [26.7–34.4]	33.1 [28.1–39.9]	30.2 [26.6–34.0]	29.4 [26.4–33.2]	<0.001
Hypertension	N (%)	188 (52.2)	42 (61.8)	70 (49.6)	76 (50.3)	0.22
Glycemia status						0.86
Normal	N (%)	176 (48.9)	33 (48.5)	72 (51.1)	71 (47.0)	
Prediabetes	N (%)	127 (35.3)	22 (32.4)	48 34.0)	57 (37.7)	
Diabetes mellitus	N (%)	57 (15.8)	13 (19.1)	21 (14.9)	23 (15.2)	
Kidney function parameters						
eGFRcr (mL/min/1.73 m^2^)	Median [IQR]	84.8 [70.4–100.2]	94.1 [78.8–103.3]	92.6 [80.4–105.8]	74.4 [63.3–87.9]	<0.001
eGFRcys (mL/min/1.73 m^2^)	Median [IQR]	89.0 [75.4–104.0]	74.0 [63.2–84.2]	93.1 [80.9–105.2]	96.2 [81.5–108.2]	<0.001
eGFRcys–eGFRcr (mL/min/1.73 m^2^)	Median [IQR]	4.0 [−5.5 to 13.4]	−17.2 [−23.1 to −12.7]	−0.8 [-4.0 to 3.6]	15.6 [11.0 to 24.3]	
uPCR >15 mg/mmol	N (%)	70 (19.8)	19 (29.7)	24 (17.1)	27 (18.0)	0.087
uACR >3 mg/mmol	N (%)	72 (20.5)	20 (31.2)	24 (17.3)	28 (18.9)	0.059

Participants are stratified by the difference between estimated glomerular filtration rate by cystatin C (eGFRcys) and creatinine (eGFRcr; >10% lower, within 10% band, >10% higher); all participants had HIV RNA <200 copies/mL.

PI, protease inhibitor; TDF, tenofovir disoproxil; uACR, urine albumin/creatinine ratio; uPCR, urine protein/creatinine ratio.

The eGFRcys was within 10% of the eGFRcr in 141 (39%), substantially lower than eGFRcr in 68 (19%), and substantially higher than eGFRcr in 151 (42%) participants. Participants for whom eGFRcys was substantially higher (median 15.6 [11.0–24.3] mL/min/1.73 m^2^) than eGFRcr were more often male, exposed to bictegravir or dolutegravir, and they had higher current CD4 cell counts, lower C-reactive protein (CRP), and lower body mass index (BMI) (Table 1). In univariable analysis, male gender, higher CD4 cell counts, and tenofovir disoproxil-sparing ART were associated with greater odds, and a higher CRP, BMI, and eGFR–creatinine with lower odds of eGFRcys being substantially higher than eGFRcr. In multivariable analysis, a higher BMI and eGFRcr remained significantly associated with lower odds of eGFRcys being substantially higher than eGFRcr (Table 2).

**TABLE 2.** Factors Associated With eGFR–Cystatin C Being >10% Higher Than eGFR–Creatinine

	Univariable			Multivariable		
	OR	95% CI	*P*	OR	95% CI	*P*
Age*	1	0.97, 1.02	0.75			
Gender (male)	1.61	1.05, 2.44	0.027	1.12	0.66, 1.90	0.67
Current CD4 cell count†	1.25	1.00, 1.60	0.058	1.14	0.89, 1.49	0.33
TDF-sparing ART	1.60	1.03, 2.52	0.039	0.92	0.53, 1.59	0.77
ART regimen including						
Efavirenz or nevirapine	ref			ref		
BTG, DTG, PI/c, or RPV	1.43	0.94, 2.18	0.098	1.12	0.67, 1.87	0.66
Current smoker	1.03	0.49, 2.12	0.93			
CRP‡	0.92	0.87, 0.97	0.006	0.94	0.87, 1.00	0.057
BMI‡	0.94	0.91, 0.98	0.001	0.95	0.91, 0.99	0.021
eGFR–creatinine§	0.59	0.51, 0.67	<0.001	0.59	0.50, 0.69	<0.001
uPCR >15 mg/mmol	0.82	0.48, 1.40	0.47			
uACR >3 mg/mmol	0.84	0.49, 1.43	0.53			

* Per year increase.

† Per 2-fold increase.

‡ Per unit increase.

§ Per 10 mL/min/1.73 m^2^ increase.

BTG, bictegravir; DTG, dolutegravir; PI/c, cobicistat-boosted protease inhibitor; RPV, rilpivirine; TDF, tenofovir disoproxil; uACR, urine albumin/creatinine ratio; uPCR, urine protein/creatinine ratio.

Of the 102 participants with eGFRcr 45–75 mL/min/1.73 m^2^, 69 (68%) had substantially higher eGFRcys than eGFRcr (median 17.8 [11.0, 29.7] mL/min/1.73 m^2^). This subgroup was also more likely to have exposure to bictegravir or dolutegravir, and less likely to have hypertension and proteinuria (Table 1, Supplemental Digital Content, http://links.lww.com/QAI/C382). In multivariable analysis, hypertension, proteinuria, and a higher eGFRcr were associated with lower odds of eGFRcys being substantially higher than eGFRcr (Table 2, Supplemental Digital Content, http://links.lww.com/QAI/C382).

Our cohort included 22 participants with eGFRcr 45–60 mL/min/1.73 m^2^. Their median eGFRcr and eGFRcys were 54 (52–56) and 65 (60–73) mL/min/1.73 m^2^, respectively; 16 (73%) had eGFRcys >60 mL/min/1.73 m^2^. All but 2 of these participants had an ACR <3 mg/mmol and thus no longer fulfilled the criteria for a CKD diagnosis.

## DISCUSSION

In individuals of African and Caribbean ancestry with well-controlled HIV and generally preserved kidney function, 42% had substantially higher eGFRcys than eGFRcr, equating to an average difference of +16 mL/min/1.73 m^2^ for eGFRcys. Substantially higher eGFRcys vs. eGFRcr was less likely to occur in those with higher CRP, higher BMI, and higher eGFRcr. Importantly, more than 60% of those with eGFRcr <60 mL/min/1.73 m^2^ no longer met the eGFR criterion for CKD when kidney function was assessed by cystatin C.

Unlike creatinine, cystatin C is not affected by muscle mass, which supports its use as a marker of kidney disease in chronic illness with muscle wasting. A higher cystatin C is associated, however, with inflammation and obesity,^16^ and restricting our analyses to individuals with well-controlled HIV aimed to limit confounding by HIV viremia. Increased cystatin C production in adipose tissue along with increased inflammation and/or metabolic dysfunction^17^ may, in part, explain the reported association between eGFRcys and mortality,^18,19^ although the relationship between inflammation, obesity, and kidney damage remains complex.^20^

Among those with eGFRcr 45–75 mL/min/1.73 m^2^, an even higher proportion (68%) was noted to have substantially higher eGFRcys than eGFRcr. Risk factors of renal impairment such as hypertension and proteinuria were associated with a lower likelihood of this, suggesting that in this stratum, a higher eGFRcys may correctly identify those without underlying kidney damage. This is supported by our data demonstrating that among those with an eGFRcr <60 mL/min/1.73 m^2^ who were reclassified as having an eGFR >60 mL/min/1.73 m^2^ with cystatin C, 91% had no albuminuria. The use of eGFRcys is particularly relevant to those with eGFRcr <75 mL/min/1.73 m^2^ who have stable kidney function and no proteinuria; in this group, both a switch in antiretroviral therapy and comorbidity-induced anxiety may be avoided if kidney function is more accurately assessed with cystatin C.^21^

A lack of association with bictegravir or dolutegravir (and other third agents that inhibit creatinine secretion) and eGFRcys being substantially higher than eGFRcr was perhaps surprising. Switch studies comparing eGFRcr and eGFRcys have noted no evolution in eGFRcys despite decline in eGFRcr after initiation of dolutegravir^22^ or dual regimens containing 1 or 2 known inhibitors of tubular transporters such as dolutegravir, cobicistat, or rilpivirine.^23^ The modest sample size and cross-sectional study design preclude analyses of individual medications in this subset or to examine prescribing bias in the setting of reduced eGFR or progressive eGFR decline that may have led to discontinuation of bictegravir or dolutegravir in those whose eGFRcr was most severely affected by these drugs.

The literature remains uncertain regarding the recommendation of eGFRcys as a marker of kidney function in people with HIV. Older data were limited and did not support a difference between eGFRcr and eGFRcys^24,25^ although these did not take into account regimens affecting tubular secretion of creatinine. More recent systematic reviews support the use of eGFRcys^26^ and suggest it performs better than eGFRcr in individuals with obesity, cancer, and HIV,^27^ and suggest that eGFRcys should be considered as an additive tool in those with an eGFRcr approximately 60 mL/min/1.73 m^2^.^7^ In addition, there is a growing body of evidence of the utility of eGFRcys in those of Black ethnicity.^15,28,29^ Data from European and North American populations suggest eGFRcr-cys, incorporating both creatinine and cystatin C, provides even greater accuracy^8,30^ although this was not confirmed in the study from sub-Saharan Africa.^5^ However, cost and lack of availability of cystatin C may limit generalized use of eGFRcys in resource-limited settings.

Strengths of this study include good representation of various Black ethnicities and women in a population with free-at-the-point-of-access health care. Dietary effects on serum creatinine were minimized by obtaining fasted blood samples, and variability in cystatin C was minimized by analysis in a central laboratory. Limitations include the cross-sectional study design, lack of other urinary biomarkers for renal dysfunction, serial creatinine data, and availability of iohexol-measured GFR or anthropometric measurements to evaluate muscle mass.

In conclusion, we report high proportions of individuals with eGFRcys substantially greater than eGFRcr, particularly among those with eGFRcr 45–75 mL/min/1.73 m^2^. These data support the use of eGFRcys for people of African and Caribbean ancestry where available, and, in particular, focused use of cystatin C to delineate individuals at higher risk of developing worsening renal function, cardiovascular disease, or mortality, or to exclude a CKD diagnosis in the absence of proteinuria.

## Supplementary Material
