# Supplementary material for: Brief Report: Cystatin C Provides Substantially Higher Glomerular Filtration Rate Estimates Than Creatinine in a Subset of Black People With HIV on Current Antiretroviral Regimens
Source: J Acquir Immune Defic Syndr. 2024 May 11;98(2):171–5. doi: 10.1097/QAI.0000000000003555 (PMC11708996; doi:10.1097/QAI.0000000000003555)
Supplement: Supplementary file 1 [file qai-98-171-s001.docx]

**Table S1 Clinical characteristics of the study participants with eGFR-creatinine 45-75 mL/min/1.73m^2^**

|  |  | | **Overall**  **(N=102)** | | **eGFRcys < eGFRcr (N=10)** | | **eGFRcys = eGFRcr (N=23)** | | **eGFRcys > eGFRcr (N=69)** | | **P-value** | |  |  |
| --- | --- | --- | --- | --- | --- | --- | --- | --- | --- | --- | --- | --- | --- | --- |
| **Demographic and HIV data** |  | |  | |  | |  | |  | |  | |  |  |
| Age | Median [IQR] | | 55.6 [50.6, 59.7] | | 56.1 [55.6, 58.0] | | 56.8 [52.8, 59.7] | | 54.3 [48.9, 59.8] | | 0.49 | |  |  |
| Gender (male) | N (%) | | 48 (47.1) | | 2 (20.0) | | 12 (52.2) | | 34 (49.3) | | 0.19 | |  |  |
| Born in African/Caribbean | N (%) | | 79 (77.5) | | 5 (50.0) | | 17 (73.9) | | 57 (82.6) | | 0.063 | |  |  |
| Time since HIV diagnosis (years) | Median [IQR] | | 14.0 [9.0, 18.0] | | 11.0 [9.0, 16.0] | | 15.0 [11.5, 20.0] | | 14.0 [9.0, 17.0] | | 0.28 | |  |  |
| Time since starting ART (years) | Median [IQR] | | 9.0 [7.0, 13.0] | | 9.0 [7.0, 13.0] | | 11.0 [7.0, 17.0] | | 9.0 [7.0, 12.8] | | 0.40 | |  |  |
| TDF-based ART regimen | N (%) | | 19 (21.6) | | 2 (25.0) | | 4 (17.4) | | 13 (22.8) | | 0.18 | |  |  |
| ART regimen by 3rd agent | N (%) | |  | |  | |  | |  | | <0.001 | |  |  |
| Efavirenz or nevirapine |  | | 21 (20.6) | | 0 (0.0) | | 4 (17.4) | | 17 (24.6) | |  | |  |  |
| Rilpivirine |  | | 10 (9.8) | | 2 (20.0) | | 2 (8.7) | | 6 (8.7) | |  | |  |  |
| Ritonavir-boosted PI |  | | 5 (4.9) | | 2 (20.0) | | 1 (4.3) | | 2 (2.9) | |  | |  |  |
| Cobicistat-boosted PI | | |  | | 19 (18.6) | | 0 (0.0) | | 8 (34.8) | | 11 (15.9) | |  | |
| Raltegravir |  | | 11 (10.8) | | 2 (20.0) | | 3 (13.0) | | 6 (8.7) | |  | |  |  |
| Bictegravir or dolutegravir |  | | 29 (28.4) | | 0 (0.0) | | 4 (17.4) | | 25 (36.2) | |  | |  |  |
| Other |  | | 7 ( 6.9) | | 4 (40.0) | | 1 (4.3) | | 2 (2.9) | |  | |  |  |
| Nadir CD4 cell count | Median [IQR] | | 161 [76, 287] | | 61 [21, 174] | | 154 [69, 327] | | 176 [90, 284] | | 0.27 | |  |  |
| Current CD4 cell count | Median [IQR] | | 548 [399, 759] | | 428 [370, 658] | | 510 [419, 827] | | 599 [399, 771] | | 0.33 | |  |  |

| **Risk factors and comorbidities** |  |  |  |  |  |  |
| --- | --- | --- | --- | --- | --- | --- |
| Smoking status | N (%) |  |  |  |  | 0.95 |
| Non-smoker |  | 80 (82.5) | 7 (77.8) | 20 (87.0) | 53 (81.5) |  |
| Ex-smoker |  | 8 (8.2) | 1 (11.1) | 1 (4.3) | 6 (9.2) |  |
| Current smoker |  | 9 (9.3) | 1 (11.1) | 2 (8.7) | 6 (9.2) |  |
| CRP (mg/L) | Median [IQR] | 3.0 [1.0, 5.0] | 3.5 [3.0, 4.0] | 3.0 [2.0, 6.0] | 3.0 [1.0, 4.85] | 0.29 |
| BMI (kg/m^2^) | Median [IQR] | 30.9 [27.3, 34.9] | 39.8 [28.7, 44.8] | 30.5 [27.0, 33.0] | 30.6 [26.9, 33.7] | 0.079 |
| Hypertension: n (%) | N (%) | 66 (64.7) | 9 (90.0) | 18 (78.3) | 39 (56.5) | 0.036 |
| Glycaemia status |  |  |  |  |  | 0.33 |
| Normal | N (%) | 45 (44.1) | 6 (60.0) | 11 (47.8) | 28 (40.6) |  |
| Pre-diabetes | N (%) | 40 (39.2) | 1 (10.0) | 9 (39.1) | 30 (43.5) |  |
| Diabetes mellitus | N (%) | 17 (16.7) | 3 (30.0) | 3 (13.0) | 11 (15.9) |  |
|  |  |  |  |  |  |  |
| **Kidney function parameters** |  |  |  |  |  |  |
| eGFRcr (mL/min/1.73m^2^) | Median [IQR] | 66.7 [60.9, 70.2] | 69.7 [64.1, 71.7] | 68.0 [63.9, 72.4] | 64.7 [60.5, 69.5] | 0.045 |
| eGFRcys (mL/min/1.73m^2^) | Median [IQR] | 75.3 [65.5, 88.0] | 54.8 [49.2, 60.4] | 67.1 [64.3, 72.2] | 83.4 [73.4, 96.1] | <0.001 |
| eGFRcys-eGFRcr (mL/min/1.73m^2^) | Median [IQR] | 11.2 [2.3, 24.8] | -13.9 [-16.0, -11.5] | 0.8 [-2.4, 2.6] | 17.8 [11.0, 29.7] |  |
| uPCR >15 mg/mmol | N (%) | 22 (21.6) | 5 (50.0) | 6 (26.1) | 11 (15.9) | 0.042 |
| uACR >3 mg/mmol | N (%) | 24 (24.0) | 5 (55.6) | 6 (26.1) | 13 (19.1) | 0.053 |

Participants are stratified by the difference between estimated glomerular filtration rate by cystatin C (eGFRcys) and creatinine (eGFRcr; >10% lower, within 10% band, >10% higher); all participants had HIV RNA <200 copies/mL.

ART=antiretroviral therapy; TDF=tenofovir disoproxil; CRP=C-reactive protein; BMI=body mass index; uPCR=urine protein/creatinine ratio; uACR=urine albumin/creatinine ratio.

**Table S2 Factors associated with eGFRcys >10% higher than eGFRcr in participants with eGFRcr 45-75 mL/min/1.73m^2^**

|  | **Univariable** | | |  | **Multivariable** | | |
| --- | --- | --- | --- | --- | --- | --- | --- |
|  | **OR** | **95% CI** | **p-value** |  | **OR** | **95% CI** | **p-value** |
| Age * | 0.96 | 0.89, 1.02 | 0.21 |  |  |  |  |
| Gender (male) | 1.32 | 0.57, 3.08 | 0.52 |  |  |  |  |
| Current CD4 cell count ** | 1.31 | 0.89, 2.11 | 0.20 |  |  |  |  |
| TDF-sparing ART | 0.96 | 0.31, 2.71 | 0.94 |  |  |  |  |
| ART regimen including |  |  |  |  |  |  |  |
| Efavirenz or nevirapine | ref |  |  |  | ref |  |  |
| BTG, DTG or PI/c | 1.97 | 0.85, 4.73 | 0.12 |  |  |  |  |
| CRP *** | 0.90 | 0.78, 1.00 | 0.081 |  | 0.94 | 0.81, 1.06 | 0.32 |
| BMI *** | 0.93 | 0.87, 0.99 | 0.027 |  | 0.94 | 0.87, 1.01 | 0.12 |
| Hypertension | 0.29 | 0.10, 0.75 | 0.015 |  | **0.30** | **0.09, 0.87** | **0.033** |
| eGFR-creatinine **** | 0.44 | 0.21, 0.86 | 0.023 |  | **0.34** | **0.14, 0.73** | **0.009** |
| uPCR >15 mg/mmol | 0.38 | 0.14, 1.00 | 0.050 |  | **0.32** | **0.10, 0.97** | **0.045** |
| uACR >3 mg/mmol | 0.45 | 0.17, 1.17 | 0.10 |  |  |  |  |

* per year increase; ** per 2-fold increase; *** per unit increase; **** per 10 mL/min/1.73m^2^ increase

Estimated glomerular filtration rate based on cystatin C (eGFRcys) and creatinine (eGFRcr); OR=odds ratio; CI=confidence interval; TDF=tenofovir disoproxil; ART=antiretroviral therapy; BTG=bictegravir; DTG=dolutegravir, PI/c=cobicistat-booted protease inhibitor; CRP=C-reactive protein; BMI=body mass index; eGFR=estimated glomerular filtration rate; uPCR=urine protein/creatinine ratio; uACR=urine albumin/creatinine ratio.
